# A Formative Assessment System in Baduanjin Physical Education Based on Inertial Measurement Unit Motion Capture

**DOI:** 10.3390/s25175423

**Published:** 2025-09-02

**Authors:** Xinyi Ma, Mingrui Shao, Xiaowei Feng, Weiping Du, Qing Yi, Puyan Chi, Hai Li

**Affiliations:** 1 School of Physical Education, Shanghai Normal University, Shanghai 201418, China; 1000569030@smail.shnu.edu.cn; 2 School of Physical Education, Hainan Normal University, Haikou 571158, China; 3 School of Physical Education, Ningxia Normal University, Guyuan 756099, China; 4 Faculty of Sports and Exercise Science, University of Malaya, Kuala Lumpur 50603, Malaysia; 5 Department of Physical Education, Shanghai Maritime University, Shanghai 201306, China; 6 College of Sport, Neijiang Normal University, Neijiang 641100, China

**Keywords:** physical education, Baduanijn, inertial measurement unit, motion capture, formative assessment

## Abstract

Traditional assessment methods in physical education often emphasize final grades, lacking real-time monitoring and feedback during the learning process. To address this limitation and enhance the formative evaluation of student performance, this study proposes a real-time assessment system for Baduanjin instruction in physical education, utilizing a commercially available inertial measurement unit-based motion capture device. The system was developed in four stages. First, a dataset was created by recruiting 20 university students and one expert physical education instructor. Participants were asked to perform standardized Baduanjin routines while wearing wireless inertial measurement unit sensors on key body joints. The collected kinematic data, sampled at 100 Hz, included joint angles and movement trajectories. Second, preprocessing and feature extraction techniques were applied to the raw data to construct a labeled dataset for training. Third, supervised machine learning algorithms were used to build models for motion type recognition and motion accuracy evaluation. Model performance was assessed using cross-validation and compared with expert evaluations. Finally, a user-facing formative assessment system was developed and tested in a controlled classroom environment. The system demonstrated a high motion recognition accuracy of 99.77%, and the correlation coefficient between system-assessed motion accuracy and expert ratings exceeded 0.80, indicating strong validity. The results demonstrate that the formative assessment system built on inertial measurement unit is appropriate for the Baduanjin physical education.

## 1. Introduction

Traditionally, educational evaluation has focused on the ultimate achievement of students at a given point in time. Formative evaluation breaks this pattern and shifts the focus to the whole process of learning. Formative assessment is a method of educational evaluation that emphasizes real-time observation, feedback, and guidance of students during the learning process [1]. Through continuous monitoring and timely feedback of students’ learning processes, formative assessment provides educators with a powerful tool to improve students’ comprehensive literacy in sports activities [2]. In physical education (PE) teaching, formative assessment is not only a tool, but also a key factor to promote students’ all-round development [3].

In the context of physical education, formative assessment serves as a dynamic and process-oriented feedback mechanism that has increasingly been recognized as a crucial approach for enhancing both student learning outcomes and instructional quality [4]. Unlike summative assessment, which focuses on evaluating final performance, formative assessment emphasizes continuous feedback, diagnosis, and instructional adjustment during the learning process [5]. This approach is particularly valuable in skill-based physical education activities, such as Baduanjin or Tai Chi, where students exhibit considerable individual variability, and final performance alone is insufficient to reflect their learning trajectories. Baduanjin is a traditional Chinese Qigong exercise that dates back to the Song Dynasty (960–1279 AD). Unlike vigorous physical exercise, Baduanjin is characterized by a low-to-moderate intensity and is suitable for individuals across age groups, including older adults and patients with chronic conditions. Each movement targets different muscle groups and is believed to promote energy flow (Qi), improve flexibility, and enhance mind–body coordination. Given its accessibility and holistic benefits, Baduanjin has been widely implemented as a non-pharmacological intervention in physical and cognitive health programs [6,7]. A complete demonstration of the Baduanjin routine can be accessed online at https://www.youtube.com/watch?v=bZhlLAoDzPA, accessed on 14 March 2023. The details of each motion are provided in manuscript-Supplementary S1. Through formative assessment, instructors can identify subtle errors in movement execution, provide timely and personalized feedback, and ultimately support students in developing body awareness, self-regulation, and autonomous learning skills. Furthermore, recent studies have demonstrated that integrating digital tools, such as wearable devices and motion capture systems, into formative assessment enhances the objectivity and precision of evaluations while also increasing students’ engagement and motivation [8].

In implementing formative assessment, various methods are employed in physical education to comprehensively understand students’ performance during physical activities. These methods include real-time observation and feedback, student self-assessment, peer evaluation, and video analysis [5,9,10]. While these approaches facilitate timely correction of students’ movements and provide guidance, they also exhibit certain limitations. Specifically, subjective assessments such as real-time observation and peer evaluation may suffer from inconsistencies in evaluation criteria, thereby compromising the objectivity of assessment [11]. Moreover, some assessment methods may be time-consuming, particularly when dealing with a large number of students, making it challenging for teachers to provide feedback and correction for each individual student’s movements [12,13]. Additionally, the utilization of technological aids is constrained by equipment limitations and insufficient school resources, resulting in some students being unable to fully benefit from such tools. Therefore, it is increasingly necessary to construct an objective, automated, and scalable movement assessment tool in physical education that can provide real-time, data-driven support for formative assessment.

The integration of digital technologies into PE teaching, such as motion capture systems, computer vision, and wearable sensors, has opened up new opportunities for formative assessment [14]. Among them, inertial measurement unit (IMU)-based motion capture technology has demonstrated high potential due to its low cost, portability, and ability to provide objective quantitative data on body movements in real-time [14,15,16]. Previous studies have applied IMU in domains such as gait analysis, rehabilitation, and athletic training to track movement patterns, assess motion quality, and provide biofeedback [17,18]. Recent studies have demonstrated the growing potential of wearable IMUs in sports and human motion analysis. Deep learning models trained on raw IMU data can achieve high accuracy in estimating motion speed, especially when sensors are placed on the lower limbs, such as the shoe [19]. Distributed IMU arrays have also been successfully applied in outdoor sports equipment testing, such as skill evaluation, enabling objective, high-frequency data collection in real-world conditions [20]. As wearable technology advances, IMUs and GPS sensors are becoming increasingly central in delivering real-time, personalized feedback in both professional and consumer sports contexts [21].

Furthermore, recent pedagogical research emphasizes that technological innovation in formative assessment must be grounded in both educational theory and subject-specific practice. Digital assessment tools can foster more individualized, inclusive, and efficient PE learning environments by enabling real-time feedback, self-regulation, and teacher support [18]. Thus, integrating IMU into PE instruction aligns with current trends in evidence-based pedagogical reform. Baduanjin, a traditional Chinese qigong routine consisting of eight standardized movements, is widely used in school and community physical education for its safety, accessibility, and benefits to both physical and mental health [22,23,24]. However, its accurate practice requires coordination of body posture, balance, and breathing rhythm—making it challenging to evaluate in large-group PE classes using subjective observation alone.

The present study aims to develop an objective movement assessment system using IMU to support formative assessment in Baduanjin-based PE. The system is designed to recognize motion sequences and evaluate motion accuracy based on predefined criteria, enabling teachers to receive accurate real-time feedback on students’ practice. This research not only contributes to the development of innovative assessment tools but also bridges modern technology and traditional physical culture. By establishing this objective assessment framework, we seek to empower physical educators with evidence-based tools to enhance teaching efficiency, support personalized learning, and improve overall instructional quality in PE.

## 2. Methods

### 2.1. Study Design and Participants

This study employed a quasi-experimental, cross-sectional design to evaluate whether an IMU-based motion capture system (Perception Neuron 2.0) can effectively differentiate Baduanjin motion accuracy across learners with different experience levels. A total of 20 undergraduate students (aged 18–22 years; 12 females and 8 males) from a university in Southwest China participated. They were assigned to either a Novice Group (*n* = 9) or an Experienced Group (*n* = 11) based on whether they had completed and passed the university’s Baduanjin course. Novice participants had no previous experience in Baduanjin and received a 30 min introductory session before motion capture. Expert grading was performed independently by two instructors with over ten years of Chinese martial arts teaching experience. All evaluation data were anonymized by replacing student names with randomly assigned participant IDs, and no identifying information was provided to the evaluators. The experts were blinded to participants’ experience levels and group assignments. Inclusion criteria required that participants had no previous formal instruction in Baduanjin, demonstrated the physical capacity to engage in low-to-moderate intensity exercise as verified through a pre-participation health screening, and provided written informed consent prior to enrolment. Participants were excluded if they presented with musculoskeletal injuries, cardiovascular disorders, or other medical conditions likely to interfere with safe participation; possessed advanced or competitive-level training experience in martial arts, Tai Chi, or Qigong; were unable to commit to the full schedule of instructional and assessment sessions; or accumulated an absence rate exceeding 20% over the course of the intervention. Ethical approval was obtained from the Research Ethics Committee of the University of Malaya (UM.TNC2/UMREC-558), and informed consent was collected from all participants.

### 2.2. Procedure

**Figure 1:**
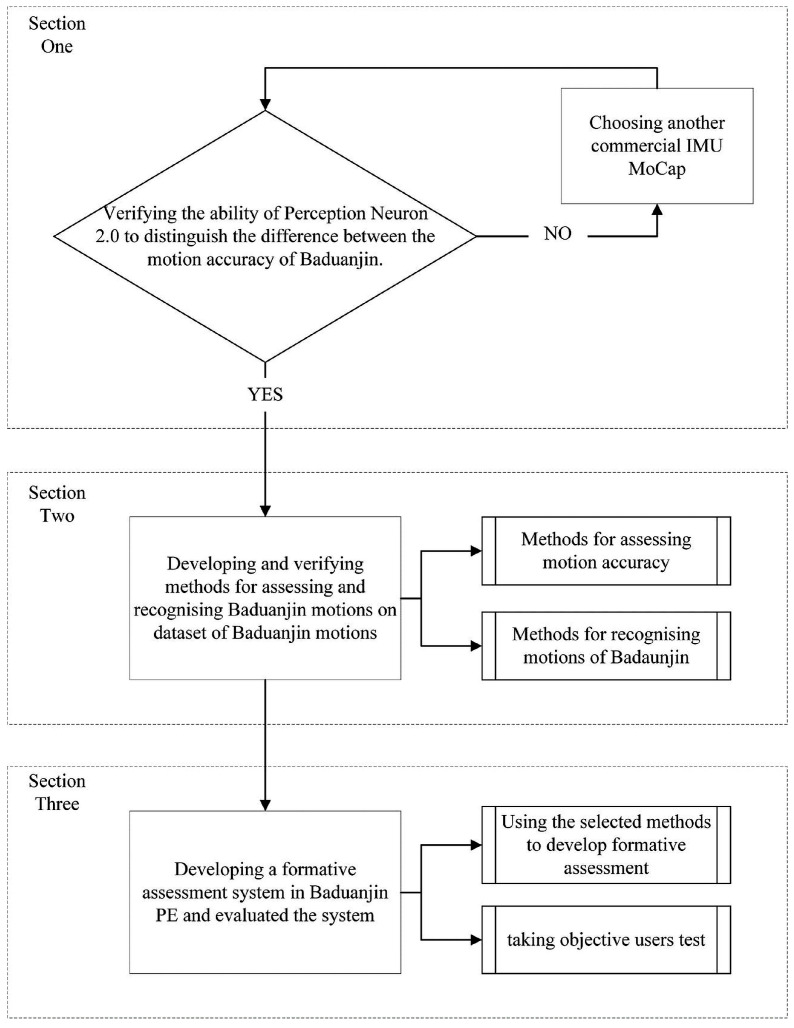
Flow diagram of the study.

This research is divided into three stages (Figure 1). Stage 1 assessed the capability of the IMU system to differentiate between motion accuracy levels. Stage 2 involved the development and verification of motion recognition methods using a labeled dataset of Baduanjin movements. Stage 3 implemented and evaluated the formative assessment system in an actual Baduanjin PE course. In section one, two groups of students with different motor accuracy levels of Baduanjin were recruited to verify the ability of IMU MoCap to distinguish the difference between the motion accuracy. In section two, methods for assessing and recognizing Baduanjin motions were developed and verified using a dataset of Baduanjin motions. Students and teachers from a university in southwestern China were recruited to create the database of Baduanjin motions. In this study, the verified methods are two commonly used types of methods for recognizing motions: sample-based and sequence-based methods. The final section applied the selected methods to develop a formative assessment system in Baduanjin PE and evaluated the system’s efficacy in Baduanjin courses. Students were recruited to test and record their Baduanjin motions while teaching Baduanjin using the built system.

### 2.3. Stage 1: Verifying IMU Effectiveness in Accuracy of Baduanjin Motions Discrimination

**Figure 2:**
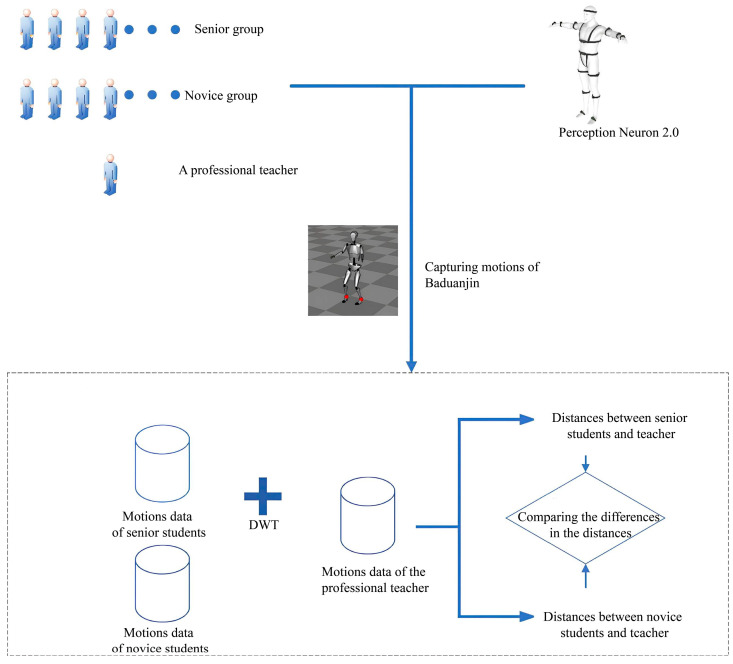
Flow diagram of verifying the effectiveness of Perception Neuron 2.0 in distinguishing motion accuracy of Baduanjin motions.

This study employed a commercial IMU system, the Perception Neuron 2.0 (Noitom Technology, Miami, FL, USA, 2018)), which has been widely used in motion capture research [25,26]. To our knowledge, no prior research has investigated the use of an IMU to distinguish the motion accuracy of Baduanjin movements. Each participant completed three motion capture trials using the Perception Neuron 2.0 system, following standardized instructions and wearing the full-body IMU suit (Figure 2). The captured data were processed to extract joint rotation data, which were then converted into quaternion format for analysis. The dynamic time warping (DTW) algorithm was used to compare student performance with the expert model, providing quantitative distance measures that represent motion accuracy.

Perception Neuron 2.0 contains multiple inertial sensor units, including a three-axis gyroscope, three-axis accelerometer, and three-axis magnetometer [27]. A total of 17 inertial sensing units were used in the investigation. The output file for the motion data captured by Perception Neuron 2.0 is the Biovision Hierarchy (BVH) file generated by the supporting software (Axis Neuron Pro, version 3.8, Noitom Technology, Miami, FL, USA)) of Perception Neuron 2.0. The BVH file format was established to store skeleton information and motion data [28]. In this research, the rotation data for each skeleton point in the BVH of motions was extracted to identify and assess motions. BVH skeletons are formed of 17 skeleton points, and rotation data is expressed in Euler angles. The rotation data was transformed into quaternions because Euler angles have gimbal lock and singularity [16,24]. All participants completed three motion captures using Perception Neuron 2.0. The novice group captured the motions immediately after 30 min of initial Baduanjin learning. The captured motion data were converted into quaternions and used dynamic time warping (DTW) to calculate the distances between the standard motions (captured from the invited teacher) and the motions of the two student groups to assess the motion accuracy of the students’ motions [18]. Since the captured motions consist of 17 skeleton points, it is necessary to calculate the distances between the corresponding skeletal points through DTW and then average the distances. In addition, considering the issue of the wrong matching by excessive time warping in DTW, the global warping window was set as 10% of the entire window span in this research to constrain the warp path to be near the diagonal of the matrix [29].

### 2.4. Stage 2: Development and Verification of Motion Recognition Methods

In the research, two common different types of methods (the sample-based and the sequence-based methods) were applied to assess the motion accuracy and recognize the motions within Baduanjin. In the sample-based methods, the features were extracted from the motion data and reduced in dimensionality to prevent data redundancy. In sequence-based methods, keyframes were extracted to prevent data redundancy and reduce processing time. Both methods were classification methods that used supervised learning based on the dataset of Baduanjin motions captured by students and teachers. Motions were assigned labels for motion accuracy or motion name. The methods used the motion data to train the classifiers based on the labels. After the model parameters of the classifier were trained, unlabeled motions could be classified. Experts in Baduanjin were invited to grade the motion accuracy of the captured motions, and then use the assessment result as labels to train the classifiers.

#### 2.4.1. Developing a Dataset of Baduanjin Motions

In this research, the dataset of Baduanjin motions was captured from undergraduate students, including 20 students and 1 professional teacher recruited in the first stage and 35 students recruited later. The dataset includes all eight standard Baduanjin motions, as described in manuscript-Supplementary S1. The second batch of recruited students carried out the Baduanjin motions once. Two professional Chinese martial art teachers with more than ten years of experience teaching Baduanjin at the university were invited to assess the motion accuracy according to the videos recorded when capturing the students’ motions. The assessment applied the grading method used in the Baduanjin course in which the motion accuracy of Baduanjin motions is divided into three grades: Fail, Pass, and Good. The Kendall test for the assessment results of the two teachers shows that the Kendall consistency of the evaluation of the eight Baduanjin movements is above 0.8, indicating that the assessment of the teachers is highly consistent.

#### 2.4.2. The Sample-Based Methods

In sample-based methods, each motion is represented by features taken from motion data. The multiple classifier models are trained on the extracted features and the labels of motions and used to classify unlabeled motions. Previous researchers have used time-domain, frequency-domain, and wavelet features to extract features to recognize motions [30,31]. This research obtained time-domain features such as mean, variance, standard deviation, skewness, kurtosis, and quartile deviation. Since the motion data comprises 17 skeleton points, the number of features extracted by one motion dataset was 17 × 3 × 6 = 306. The extracted features need to be normalized and reduced dimensionality for subsequent training models. The extracted features were normalized to the range [0, 1], and Principal component analysis was used to reduce the dimensionality of features [32]. Based on the features and labels of motions, the classifiers, including *k*-Nearest Neighbor (*k*-NN), Support Vector Machines (SVM), Naive Bayes (NB), Logistic Regression, Decision Tree (DT), Back Propagation neural network (BPNN), Radial basis function neural network (RBFNN) and One-dimensional CNN (1D-CNN) were trained to assess and recognize motions. The sample-based methods involved in this research were constructed and verified using Matlab 2020b as the platform.

#### 2.4.3. The Sequence-Based Methods

The difference from sample-based methods is that frequency-based methods do not extract features but analyze motion data on quaternions as time-series data. Considering the limited storage space and bandwidth capacity available to users in teaching, extracting keyframes is used to reduce motion data to improve application adaptability. The study used k-means clustering to extract keyframes corresponding to the compression rate using a preset compression rate [33]. In order to confirm the efficacy of extracting keyframes and obtain a reasonable compression ratio, the interpolation method was applied for reconstructing motions, calculating motion reconstruction error, and then setting the extraction compression ratio to 15% [34,35]. Based on the keyframes and labels of motions, the sequence-based methods, including DTW combined with classifiers (*k*-NN, SVM, NB, Logistic Regression, DT, BPNN, and RBFNN), Hidden Markov Model (HMM), and Long Short-Term Memory (LSTM), Bidirectional LSTM (BiLTSM), and Gated Recurrent Units (GRUs), were applied to train the models to assess and recognize motions. The frequency-based methods involved in this research were constructed and verified using Matlab 2020b as the platform.

### 2.5. Stage 3: Developing a Formative Assessment System and Taking Objective User Test

The objective user test was conducted to evaluate the effectiveness of the developed formative assessment system in Baduanjin PE and to examine whether the implemented motion assessment and recognition methods could be applied in a teaching context. Undergraduate students with no prior experience in Baduanjin, no physical disabilities, and no clinical or mental illnesses were recruited. All participants completed the eight-week Baduanjin PE curriculum, which comprised eight lessons. After each lesson, students were required to repeat the movements learned in that session. Their performance was evaluated both by the course instructor using traditional manual assessment and by the formative assessment system. The evaluation considered two aspects: the accuracy of each individual motion and the integrity of the motion sequence, including missing motions and sequence errors. This process was repeated after every lesson, and the results from the instructor and the system were compared at the end of the course to assess the system’s validity.

The formative assessment system was designed as a general framework for assessing motion accuracy and detecting missing or incorrectly ordered motions. The framework supports the integration of different classification algorithms; in the present implementation, the system served as a platform to test motion analysis methods without claiming optimality for any specific model. Further comparative studies with alternative algorithms will be undertaken to determine the most effective approach for this application. The system processes IMU-captured motion data in a continuous workflow. Motion data in BVH file format are imported, skeletal information is extracted, and joint rotation data are converted into quaternion format. Feature values are then computed and reduced in dimensionality using principal component analysis to minimize redundancy. The chosen classification algorithm is applied to assess individual motion accuracy and to recognize motion types. Recognized sequences are compared with a reference sequence to detect missing motions or ordering errors, and the results are output for further analysis and instructional feedback.

### 2.6. Statistical Analysis

Based on the calculated distances, IBM SPSS Statistics 25.0, using independent samples T-test for normally distributed data and Mann–Whitney U test for normally distributed data, was used as a platform to evaluate the significance of the differences in motion accuracy between motions

## 3. Results

### 3.1. Results of Accuracy Discrimination of Baduanjin Exercise

Baduanjin comprises eight standardized movements. In this study, each movement was recorded three times from 20 students and 1 instructor, producing 63 motion datasets. Using the instructor’s performance as the reference, dynamic time warping (DTW) was applied to calculate the distance between each student and the instructor at 17 skeletal points, representing movement accuracy. For Motions 2, 3, and 4 (independent-samples t-tests, Table 1), novice students showed significantly greater DTW distances than experienced students, indicating lower movement accuracy. Specifically, for Motion 2, novices (M = 640.76, SD = 74.38) scored higher than seniors (M = 565.72, SD = 61.64), *t* (58) = 4.28, *p* < 0.001. For Motion 3, novices (M = 543.46, SD = 78.92) again scored higher than seniors (M = 455.75, SD = 54.30), *t* (58) = 5.09, *p* < 0.001. For Motion 4, novices (M = 536.45, SD = 41.44) scored higher than seniors (M = 468.66, SD = 47.70), *t* (58) = 5.81, *p* < 0.001. 

For Motions 1, 5, 6, 7, and 8 (Mann–Whitney U tests, Table 2), significant differences were also observed, with experienced students achieving better accuracy in every case. Motion 1 yielded U = 229.00, Z = −3.22, *p* = 0.001; Motion 5, U = 136.00, Z = −4.60, *p* < 0.001; Motion 6, U = 299.00, Z = −2.18, *p* = 0.029; Motion 7, U = 259.00, Z = −2.77, *p* = 0.006; and Motion 8, U = 130.00, Z = −4.69, *p* < 0.001. Across all eight movements, experienced students consistently demonstrated smaller DTW distances from the instructor’s performance, confirming that the Perception Neuron 2.0 motion data can effectively differentiate performance accuracy levels in Baduanjin practice.

### 3.2. Results of Assessing and Recognizing Motions on the Developed Methods

Using the established motion dataset and the motion scores from two professional Chinese martial arts instructors (Teacher A and Teacher B) as labels, the classification accuracies of different methods under 10-fold cross-validation are presented in Table 3 and Table 4. For Teacher A (Table 3), the highest accuracy for Motion 1 was achieved by the sequence-based DTW + k-NN method (94.74%), and for Motion 7 by the sample-based SVM method (95.79%); for the remaining six motions, the sample-based k-NN method produced the highest accuracy. For Teacher B (Table 4), the sample-based k-NN method achieved the highest accuracy for six of the eight motions, with only Motion 1 and Motion 7 showing different best-performing methods. Overall, while no single method achieved the top accuracy across all motions, the sample-based k-NN method consistently outperformed other approaches for the majority of movements.

From Table 5, the processing time of the sample-based *k*-NN method is the shortest among the validated methods (0.008 s). Therefore, considering the accuracy and processing time comprehensively, the method for assessing motion accuracy chose the sample-based *k*-NN method. Six methods recognized motions with over 99% accuracy. They were SVM (99.47%), Logistics regression (99.21%) and 1D-CNN (99.74%) in the sample-based methods and DTW + *k*-NN (99.47%), DTW + SVM (99.61%), and HMM (99.08%) in the sequence-based methods. However, the processing times of the six methods varied widely. The sample-based 1D-CNN method with the highest accuracy took 80.958 s, but the sample-based SVM method took 0.914 s.

The Chi-square test assessed the significant differences in recognizing motions of the six methods on the current experimental test results, and the results show there are no significant differences (Table 6). Therefore, considering the accuracy and processing time comprehensively, the method we chose for recognizing motions was the sample-based SVM method.

### 3.3. Results of the Objective User Test

The Baduanjin PE course comprised eight lessons delivered over eight weeks, with one lesson per week. The teaching schedule progressed as follows: Lesson 1 introduced Motions 1–3; Lessons 2 and 3 provided practice for Motions 1–3; Lesson 4 introduced Motions 4–6; Lesson 5 provided practice for Motions 1–6; Lesson 6 introduced Motions 7–8; and Lessons 7 and 8 provided practice for the complete set of eight motions. As learning and practice were scheduled in different weeks, not every lesson involved new movements. Over the course, each student performed eight repetitions of Motion 1, eight repetitions of Motion 2, and eight repetitions of Motion 3 (in Lessons 1–8). Motions 4, 5, and 6 were each performed five times (in Lessons 4–8), while Motions 7 and 8 were each performed three times (in Lessons 6–8). In total, 45 motion samples were recorded for each student across the eight-week course.

Although a total of 450 motion data points (45 data sets × 10 students = 450 data points) could have been obtained to verify the established formative assessment system, only 439 motions data were recorded because some students missed the motions in the learning process. It was found that the developed system wrongly recognized Motion 5 as Motion 3 for one student. In general, the recognition rate was still excellent, reaching 99.77%. There are four missing motions in the data recorded for Motion 3 for three students. Student ID 1 and 4 had one missing motion (Lesson 4), and Student ID 3 had two missing motions (Lessons 4 and 5). All three missing motions happened in Lesson 4 when students learned new motions. The formative assessment system can output the captured motions of any particular student to analyze errors in the sequence of motions and missing motions. Student ID 1 completed all motion assessments for the first three lessons. However, after learning the new motions (Motion 4 to Motion 6) in Lesson 4, the student missed Motion 3 in the assessment. Moreover, after learning the new motion (Motion 7 and Motion 8) in Lesson 6, the student missed Motion 6 in the assessment. The phenomenon of “forgetting a motion” reappeared in Lesson 4 and Lesson 6.

The effectiveness of the developed formative assessment system was evaluated by comparing its grading results with those of the instructor, as shown in Table 7. Motion assessments were classified into three levels: Fail, Pass, and Good. Across all eight Baduanjin movements, the system’s evaluations showed high agreement with the instructor’s, with Kendall’s correlation coefficients exceeding 0.80 for every motion (range: 0.835–0.904). For Motion 1, the system recorded 14 Fail, 58 Pass, and 8 Good ratings, closely matching the instructor’s 16 Fail, 58 Pass, and 6 Good ratings (Kendall’s τ = 0.904). A similar consistency was observed for Motion 2 (system: 6/52/22; instructor: 5/58/17; τ = 0.865), Motion 3 (9/28/39 vs. 9/35/32; τ = 0.855), Motion 4 (6/22/18 vs. 7/25/14; τ = 0.867), Motion 5 (4/26/20 vs. 5/29/16; τ = 0.862), Motion 6 (5/31/11 vs. 7/32/8; τ = 0.835), and Motion 7 (3/22/5 vs. 3/24/3; τ = 0.850). For Motion 8, no Fail ratings were recorded by either the system or the instructor. According to the instructors, this is the simplest movement in Baduanjin and can be mastered in a short time, though long-term practice is still required to achieve high accuracy. For this motion, only Pass and Good ratings were assigned, with identical counts from the system (15/15) and the instructor (15/15), yielding a Kendall’s τ = 0.867. These results confirm that, in the objective user test, the formative assessment system can reliably evaluate the accuracy of students’ Baduanjin performances, producing grading outcomes highly consistent with expert judgment.

## 4. Discussion and Implications

This study aimed to address a pressing challenge in Chinese physical education—the difficulty of conducting formative assessment in large-class settings, particularly for traditional Chinese sports such as Baduanjin. The results demonstrated that commercial IMU-based motion capture, specifically Perception Neuron 2.0, could effectively distinguish between students of differing skill levels in terms of motion accuracy. The verification stage showed statistically significant differences in DTW-based distance metrics between novices and experienced practitioners, affirming the feasibility of using this technology to support formative assessments in physical education.

The results of Section One show significant differences in the distance between the two groups of students’ motions and the teacher’s motions that verify that the motion data captured by the chosen commercial IMU MoCap: Perception Neuron 2.0 could effectively distinguish Baduanjin motions with different motion accuracy (Table 1 and Table 2). Based on the verification results from Section One, Section Two developed and selected the appropriate methods for assessing motion accuracy and recognizing the motions of Baduanjin. Two different types of methods (sample-based and sequence-based methods) were applied to assess movement accuracy and recognize the motions of Baduanjin. Using the built dataset of Baduanjin motions, the results show that the sample-based *k*-NN method was selected for assessing motion accuracy for high accuracy and short processing time. In recognizing motions, although there were several methods with accuracy over 99%, there is no significant difference in the chi-square test between the methods in the current results. The sample-based SVM method was selected for recognizing motions considering the processing time. The findings clearly indicate that experienced students performed Baduanjin movements with significantly greater accuracy than novices. This aligns with previous research on skill acquisition in traditional and modern sports, which shows that experienced practitioners typically display smoother kinematic trajectories, reduced intra-movement variability, and enhanced postural control. Our results extend these observations to the context of Baduanjin and underscore that the level of practice and familiarity plays a key role in improving biomechanical execution. Moreover, the system successfully identified specific student learning difficulties—such as forgetting previously learned motions during the acquisition of new ones—highlighting its potential for real-time educational intervention. The formative assessment system in Baduanjin PE was developed based on the optimal assessment of motion accuracy and recognizing motion methods selected by the verification methods. Moreover, the objective user test of the system was carried out. The objective user test results show that the accuracy of the formative assessment system in the motion recognition of students reaches 99.77%. The consistency test (Kendall test) of the formative assessment system and teacher on assessing the motion accuracy of students exceeds 0.8. These objective user test results show that the developed formative assessment system effectively assesses motion accuracy and recognizes motions. In addition, using the formative assessment system, problems students face in the learning process can be detected immediately. For example, using recognizing the motions of students as a metric, the system shows the problem of forgetting motions that often occur when learning Baduanjin. The system detected three students who forgot Motion-3 in learning Baduanjin in Lesson 4. Lesson 4 requires students to learn new motions, which leads some students to forget the previously learned motions when learning the new motions. This phenomenon is seen for Student ID 1. The formative assessment system shows that the student was unable to remember Motion 3 (during Lesson 4) and Motion 6 (during Lesson 6). Lastly, the developed formative assessment system could trace the learning process. As an example, for Student ID 1, the recorded result clearly shows the learning progress of all the motion accuracy throughout the eight-week learning process. Therefore, the objective user test results reflect that the first-generation formative assessment system can assess students in the learning process to discover the mistakes made by students.

Formative assessment refers to the assessment to discover the problems of students during the learning process, and it usually consists of a small number of items but requires frequent measurement. Formative assessment can assess how well students are progressing and provide teachers with important information about managing instruction. In contrast, summative assessment does not consider the development of students and problems in the process of learning and feedback from teachers [3]. While previous research on formative assessment tools has primarily focused on mainstream sports or dynamic activities, this study is one of the few to validate such technologies in slow, meditative traditional Chinese exercises. Compared to yoga, Tai Chi, or martial arts, which have also benefited from similar analyses, Baduanjin poses unique challenges due to its subtle motion characteristics and emphasis on internal flow. Despite this, our approach demonstrated similar efficacy in quantifying motion accuracy and identifying learning gaps. This reinforces the argument that motion tracking technologies are not limited to high-intensity sports and can be effectively adapted to diverse physical disciplines. Similar trends have been observed in studies on other sports, such as Tai Chi, yoga, or martial arts: experienced practitioners typically demonstrate smoother joint trajectories, reduced movement variability, and better postural stability [36,37]. Our study provides additional empirical support that motion capture-based assessments can distinguish between skill levels not only in dynamic or competitive sports but also in slow, meditative forms of exercise like Baduanjin. This contributes to the growing literature on the quantification of traditional Chinese exercises using wearable technology.

The results of this study hold significant implications for the field of physical education. Firstly, the successful development of a formative assessment system capable of real-time monitoring of student movements and accurate evaluation of motion accuracy, achieved through the use of commercial IMU technology, provides physical education teachers with an effective tool. This tool enables them to better understand students’ performance during the learning process, promptly identify issues, and offer personalized guidance and support. This contributes to enhancing teaching quality and enables students to develop their physical literacy more comprehensively. Secondly, the study results demonstrate the immense potential of commercial IMU technology in the context of traditional Chinese physical education. Despite limited research in this area, the study validates the effectiveness of this technology in recognizing motion accuracy. This provides a reliable theoretical basis for future efforts to promote the use of IMU MoCap technology in traditional Chinese physical education. Furthermore, the study confirms the practicality and feasibility of the formative assessment system. Through objective user testing, the system demonstrates excellent performance in student motion recognition and high consistency with teacher evaluations. This indicates that the system can serve as an effective tool to help teachers manage classrooms, provide personalized guidance to students, and promptly identify and address issues encountered during the learning process. Practical implications of this study extend to physical education and health promotion. The ability to objectively assess movement quality using wearable sensors offers a promising tool for PE teachers to provide personalized instruction and track student progress. Additionally, the framework developed here may be adapted for other sports or rehabilitation programs where form and accuracy are crucial. For example, similar motion evaluation systems could be applied in yoga, gymnastics, or elderly balance training, aligning well with current trends in digitized, data-driven sport pedagogy [38,39].

Although the present study focused on Baduanjin movements, the proposed IMU-based formative assessment framework is not limited to this discipline. With appropriate adjustments to the reference motion library and accuracy criteria, the system could be applied to assess other structured movement practices, such as Tai Chi, yoga, and martial arts, as well as technical skills in sports like gymnastics, golf, and swimming. Beyond sports and traditional exercises, the approach could be extended to rehabilitation and physical therapy settings to monitor patients’ progress, provide objective feedback, and support remote or home-based training. These examples illustrate the potential for broader application of this technology in movement assessment and skill acquisition monitoring across diverse domains.

This study has several limitations that should be considered when interpreting the findings. The sample consisted of full-time students from a single institution, which may limit the generalizability of the results to other populations. Future research should recruit participants from a wider range of age groups, backgrounds, and physical ability levels to improve external validity. The IMU-based assessment system was tested in a controlled indoor setting, and its performance under different environmental conditions was not evaluated. Further studies should assess its reliability and accuracy in varied real-world contexts, such as outdoor classes or settings with limited space. The evaluation framework focused primarily on kinematic accuracy and did not incorporate physiological or perceptual measures, which could provide a more comprehensive understanding of learning outcomes. Future work could integrate motion data with physiological indicators and self-reported measures. Finally, while the system demonstrated potential for enhancing formative assessment, the need for technical setup and calibration may hinder large-scale adoption in schools with limited resources. Streamlining system deployment and automating data processing should be priorities for future development to support broader implementation.

## 5. Conclusions

The study demonstrates that integrating commercial IMU technology into formative assessment practices in PE holds promise for improving teaching and learning outcomes. The developed formative assessment system effectively monitors student motion in real-time and accurately evaluates motion accuracy. By leveraging technology in assessment, educators can gain valuable insights into student performance and provide timely guidance and support. This research contributes to advancing assessment methods in physical education and underscores the potential of technology in enhancing teaching practices and student learning experiences.

## Figures and Tables

**Table 1:** Differences in motion accuracy between novice and senior students (independent sample T-test).

Motion	Group	N	Mean	Std. M	F	Sig.	t	Sig. ^a^
Motion-2	N.S	27	640.76	74.38	2.29	0.14	4.28	0.000
	S.S	33	565.72	61.64			4.20	0.000
Motion-3	N.S	27	543.46	78.92	4.88	0.03	5.09	0.000
	S.S	33	455.75	54.30			4.90	0.000
Motion-4	N.S	27	536.45	41.44	0.06	0.81	5.81	0.000
	S.S	33	468.66	47.70			5.89	0.000

^a^ 2-tailed; N.S, novice students; S.S, senior students.

**Table 2:** Differences in motion accuracy between novice and senior students (Mann–Whitney U test).

Motion	Group	N	Mean Rank	Sum of Ranks	M-W U ^a^	Wilcoxon	Z	Asymp. Sig. ^b^
Motion-1	N.S	27	38.52	1040.00	229.00	790.00	−3.22	0.001
	S.S	33	23.94	790.00				
Motion-5	N.S	27	41.96	1133.00	136.00	697.00	−4.60	0.000
	S.S	33	21.12	697.00				
Motion-6	N.S	27	35.93	970.00	299.00	860.00	−2.18	0.029
	S.S	33	26.06	860.00				
Motion-7	N.S	27	37.41	1010.00	259.00	820.00	−2.77	0.000
	S.S	33	24.85	820.00				
Motion-8	N.S	27	42.19	1139.00	130.00	691.00	4.69	0.000
	S.S	33	20.94	691.00				

^a^ Mann–Whitney U; ^b^ 2-tailed; N.S, novice students; S.S, senior students.

**Table 3:** Accuracy of assessing motion accuracy on different methods using the scores of Teacher A as labels.

Methods		Accuracy (%)							
		1 ^a^	2	3	4	5	6	7	8
Sample-based	k-NN	89.47	92.63 ^b^	91.58 ^b^	92.63 ^b^	89.47 ^b^	92.63 ^b^	87.37	88.42 ^b^
	SVM	89.47	84.21	80.00	92.63	80.00	75.79	95.79 ^b^	80.00
	NB	81.05	83.16	74.74	90.53	77.89	80.00	82.11	76.84
	LR	78.95	71.58	62.11	81.05	84.21	77.89	81.05	76.84
	DT	73.68	65.26	65.26	61.05	65.26	62.11	73.68	65.26
	BPNN	73.68	61.05	63.16	70.53	78.95	66.32	81.05	73.68
	RBFNN	83.16	67.37	75.79	75.79	78.95	75.79	84.21	70.53
	1D-CNN	71.58	76.84	69.47	76.84	88.42	91.58	78.95	74.74
Sequence-based	DTW + k-NN	94.74 ^b^	86.32	77.90	80.00	84.21	77.90	87.37	85.26
	DTW + SVM	66.32	62.11	69.47	74.74	63.16	65.26	69.47	78.95
	DTW + NB	77.90	72.63	74.74	84.21	65.26	70.53	70.53	74.74
	DTW + LR	66.32	63.16	67.37	73.68	63.16	63.16	66.32	74.74
	DTW + DT	69.47	63.16	82.11	70.53	67.37	68.42	74.74	69.47
	DTW + BPNN	85.26	71.58	71.58	73.68	66.32	67.37	69.47	84.21
	DTW + RBFNN	89.47	84.21	72.63	75.79	80.00	81.05	82.11	83.16
	HMM	84.21	80.00	78.95	90.53	76.84	78.95	83.16	77.90
	LSTM	75.79	77.90	82.11	84.21	72.63	84.21	78.95	78.95
	BiLSTM	84.21	80.00	78.95	90.53	76.84	78.95	83.16	77.90
	GRU	80.00	75.79	67.37	83.16	74.74	81.05	82.11	72.63

Note: ^a^ Motion; ^b^ The highest accuracy. Dynamic time warping (DTW), k-Nearest Neighbor (k-NN), Support Vector Machines (SVM), Naive Bayes (NB), Logistic Regression (LR), Decision Tree (DT), Back Propagation neural network (BPNN), Radial basis function neural network (RBFNN), One-dimensional CNN (1D-CNN), Hidden Markov Model (HMM), Long Short-Term Memory (LSTM), Bidirectional LSTM (BiLTSM), and Gated Recurrent Units (GRU).

**Table 4:** Accuracy of assessing motion accuracy on different methods using the scores of Teacher B as labels.

Methods		Accuracy (%)							
		1 ^a^	2	3	4	5	6	7	8
Sample-based	k-NN	89.47	86.32 ^b^	88.42 ^b^	91.58 ^b^	91.58 ^b^	86.32 ^b^	85.26	86.32 ^b^
	SVM	83.16	72.63	74.74	86.32	83.16	84.21	87.37 ^b^	73.68
	NB	78.95	78.95	68.42	88.42	80.00	81.05	84.21	75.79
	LR	78.95	71.58	62.11	81.05	84.21	77.89	81.05	76.84
	DT	73.68	65.26	65.26	61.05	65.26	62.11	73.68	65.26
	BPNN	73.68	61.05	63.16	70.53	78.95	66.32	81.05	73.68
	RBFNN	83.16	67.37	75.79	75.79	78.95	75.79	84.21	70.53
	1D-CNN	72.63	62.11	76.84	80.00	87.37	81.05	78.95	72.63
Sequence-based	DTW + k-NN	92.63 ^b^	77.89	77.90	80.00	83.16	83.16	86.32	83.16
	DTW + SVM	66.31	60.00	69.47	69.47	66.32	65.26	72.63	77.90
	DTW + NB	75.79	73.68	71.58	71.58	74.74	75.79	74.74	67.37
	DTW + LR	64.21	61.05	61.05	68.42	61.05	62.11	70.53	71.58
	DTW + DT	67.37	62.11	60.00	70.53	76.84	76.84	73.68	81.05
	DTW + BPNN	68.42	62.11	66.32	65.26	66.32	54.74	71.58	78.95
	DTW + RBFNN	78.95	74.74	76.84	62.11	86.32	78.95	86.32	83.16
	HMM	83.16	73.68	80.00	80.00	77.90	76.84	82.11	85.26
	LSTM	76.84	71.58	77.90	75.79	76.84	82.11	84.21	82.11
	BiLSTM	82.11	75.79	78.95	74.74	76.84	82.11	83.16	85.26
	GRU	76.84	67.37	69.47	71.58	75.79	78.95	77.90	88.42

Note: ^a^ Motion; ^b^ The highest accuracy. Dynamic time warping (DTW), k-Nearest Neighbor (k-NN), Support Vector Machines (SVM), Naive Bayes (NB), Logistic Regression (LR), Decision Tree (DT), Back Propagation neural network (BPNN), Radial basis function neural network (RBFNN), One-dimensional CNN (1D-CNN), Hidden Markov Model (HMM), Long Short-Term Memory (LSTM), Bidirectional LSTM (BiLTSM), and Gated Recurrent Units (GRU).

**Table 5:** Accuracy and processing time of different methods for assessing and recognizing motions.

Methods		Accuracy (%)	Assessing (Seconds)	Recognizing (Seconds)
Sample-based	k-NN	97.63	0.008 ^a^	0.055 ^b^
	SVM	99.47	4.751	0.914
	NB	97.89	0.021	0.174
	LR	99.21	0.020	0.407
	DT	84.47	0.010	0.087
	BPNN	86.97	7.709	13.270
	RBFNN	75.53	0.063	0.295
	1D-CNN	99.74 *	9.179	80.958
Sequence-based	DTW + k-NN	99.47	3.810	3.823
	DTW + SVM	99.61	4.119	6.909
	DTW + NB	91.84	4.057	6.757
	DTW + LR	94.21	4.382	10.163
	DTW + DT	93.68	3.947	4.809
	DTW + BPNN	91.05	14.830	24.665
	DTW + RBFNN	75.79	3.898	5.439
	HMM	99.08	4.119	61.144
	LSTM	96.45	14.132	123.477
	BiLSTM	97.37	27.995	239.190
	GRU	97.50	11.943	106.513

Note: * The highest accuracy; ^a^ minimum processing time. ^b^ Minimum processing time. dynamic time warping (DTW), k-Nearest Neighbor (k-NN), Support Vector Machines (SVM), Naive Bayes (NB), Logistic Regression (LR), Decision Tree (DT), Back Propagation neural network (BPNN), Radial basis function neural network (RBFNN), One-dimensional CNN (1D-CNN), Hidden Markov Model (HMM), Long Short-Term Memory (LSTM), Bidirectional LSTM (BiLTSM), and Gated Recurrent Units (GRU).

**Table 6:** Chi-square test on the methods for recognizing motions with over 99% accuracy.

Methods		Recognized Motions		Total
		Correct	Incorrect	
Sample-based	SVM	756	4	760
	Logistics regression	754	6	760
	1D-CNN	758	2	760
Sequence-based	DTW + k-NN	756	4	760
	DTW + SVM	757	3	760
	HMM	753	7	760
Value of Pearson Chi-Square		4.023 ^a^		
Asymptotic Significance (2-sided) of Pearson Chi-Square		0.546		

^a^ Five cells (50.0%) have an expected count of less than 6. The minimum expected count is 4.33.

**Table 7:** The assessment results and consistency analysis (Kendall test) between the system and teacher.

Motion ^a^	System			Teacher			Kendall Value
	Fail	Pass	Good	Fail	Pass	Good	
1	14	58	8	16	58	6	0.904
2	6	52	22	5	58	17	0.865
3	9	28	39	9	35	32	0.855
4	6	22	18	7	25	14	0.867
5	4	26	20	5	29	16	0.862
6	5	31	11	7	32	8	0.835
7	3	22	5	3	24	3	0.850
8	NA	15	15	NA	15	15	0.867

^a^ Motion-1.

## Data Availability

The datasets used and/or analyzed during the current study are available from the corresponding author on reasonable request.

## Supplementary Materials

The following supporting information can be downloaded at: https://www.mdpi.com/article/10.3390/s25175423/s1, Supplementary S1: The standard Baduanjin routine consists of eight sequential movements.
