# Supplementary material for: A Formative Assessment System in Baduanjin Physical Education Based on Inertial Measurement Unit Motion Capture
Source: Sensors (Basel). 2025 Sep 2;25(17):5423. doi: 10.3390/s25175423 (PMC12430958; doi:10.3390/s25175423)

The standard Baduanjin routine consists of eight sequential movements:

Motion 1 – Two Hands Hold up the Heavens: Stretching upward with both hands to stimulate the triple burner meridian.

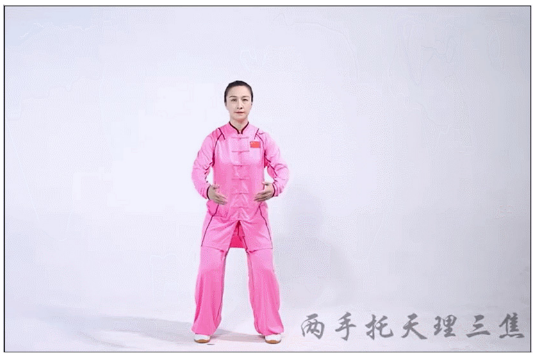

Motion 2 – Drawing the Bow to Shoot the Hawk: Simulating a bow-drawing posture to expand the chest and strengthen upper limb muscles.

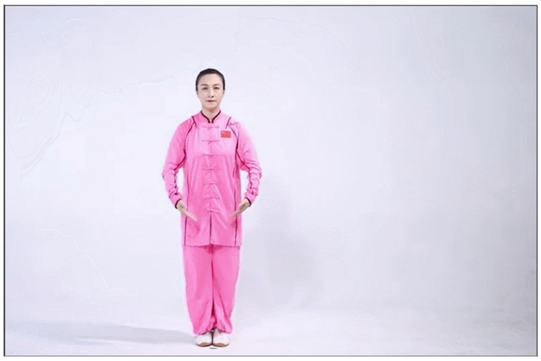

Motion 3 – Separate Heaven and Earth: One hand pushing upward and the other downward to regulate internal organs.

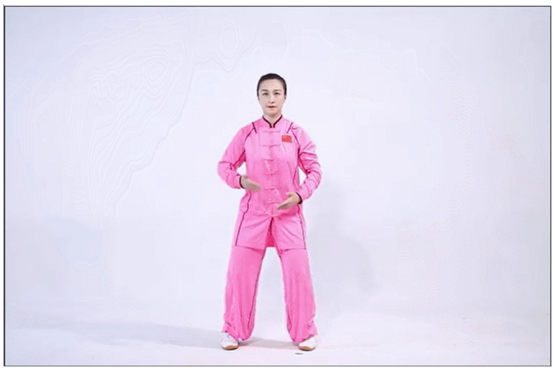

Motion 4 – Wise Owl Gazes Backwards: Turning the head to look behind to improve cervical spine flexibility.

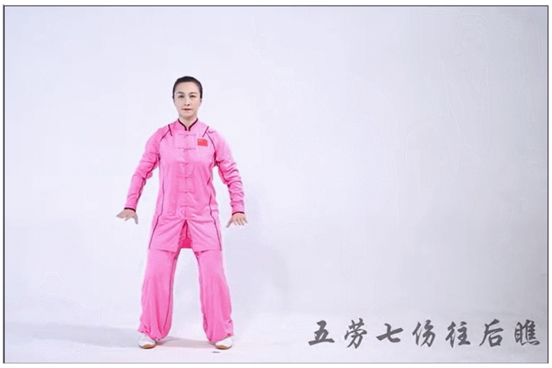

Motion 5 – Sway the Head and Shake the Tail: Coordinated upper and lower body twisting to regulate the heart and lungs.

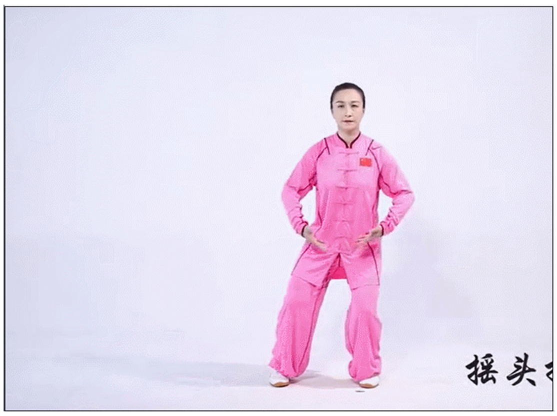

Motion 6 – Two Hands Hold the Feet: Forward bending to stretch the spine and hamstrings.

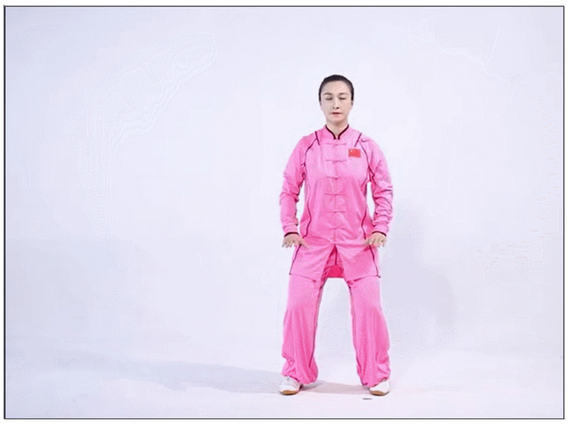

Motion 7 – Clench the Fists and Glare Fiercely: Punching with force to enhance muscle strength and focus.

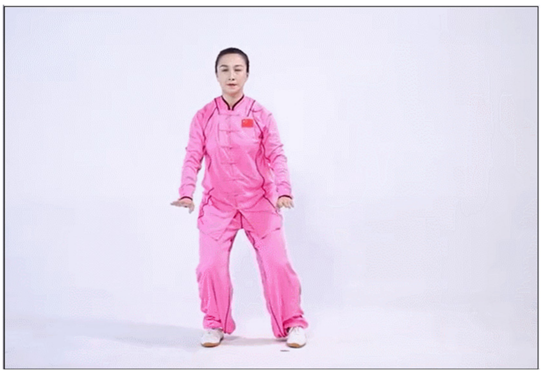

Motion 8 – Bounce on the Toes: Repeated heel raises to promote circulation and strengthen the lower limbs.

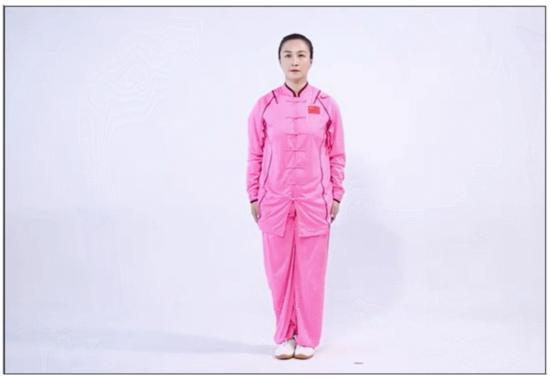

Supplement: Supplementary file 1 [file sensors-25-05423-s001.zip › sensors-3741115-supplementary.pdf]
